# Understanding Severe Sleep-Disordered Breathing in Down Syndrome: Insights from a Clinical–Polysomnographic Cohort

**DOI:** 10.3390/jcm15145581

**Published:** 2026-07-16

**Authors:** Marco Zaffanello, Luca Levrini, Elena De Giorgi, Alessandra Greta Grassi, Daniela Simoncini, Maddalena Marinoni, Stefano Saran, Massimo Agosti, Luana Nosetti

**Affiliations:** 1 Department of Surgical Sciences, Dentistry, Gynaecology and Pediatrics, University of Verona, 37129 Verona, Italy; 2 Department of Human Science and Innovation for the Territory, University of Insubria, 21100 Varese, Italy; luca.levrini@uninsubria.it (L.L.); stefanosaran@yahoo.com (S.S.); 3 Division of Paediatrics, Insubria University, “Filippo del Ponte” Hospital, 21100 Varese, Italy; edegiorgi@studenti.uninsubria.it (E.D.G.); aggrassi@studenti.uninsubria.it (A.G.G.); daniela.simoncini@asst-settelaghi.it (D.S.); massimo.agosti@asst-settelaghi.it (M.A.); luana.nosetti@uninsubria.it (L.N.); 4 Pediatric Onco-Haematology Unit, ASST-Settelaghi, “Filippo del Ponte” Hospital, 21100 Varese, Italy; maddalena.marinoni@asst-settelaghi.it

**Keywords:** adenoid hypertrophy, children, Down syndrome, obstructive sleep apnoea, oxygen desaturation, polysomnography, sleep-disordered breathing

## Abstract

**Introduction**: Children with Down syndrome (DS) are at increased risk of sleep-disordered breathing (SDB), but clinical correlates of polysomnography-derived severity remain incompletely defined. **Objectives**: This retrospective clinical–polysomnographic cohort study examined associations between selected demographic, anthropometric, cardiac, and otolaryngological variables and SDB severity metrics in children and adolescents with DS. **Materials and Methods**: Forty-eight participants aged ≤ 18 years underwent overnight polysomnography for suspected SDB. Respiratory events were scored according to paediatric AASM criteria; hypopnoeas required a ≥30% airflow reduction associated with ≥3% oxygen desaturation and/or arousal, and ODI was calculated using ≥3% desaturation events per hour of total sleep time. Outcomes included Apnoea–Hypopnoea Index (AHI), ODI, minimum SpO_2_, and time with SpO_2_ < 90%. **Results**: OSA was highly prevalent: AHI ≥ 1 event/h was observed in 93.8% of participants, AHI ≥ 5 events/h in 58.3%, and AHI ≥ 10 events/h in 35.4%. Mean AHI was 10.51 ± 11.82 events/h, and mean ODI was 7.07 ± 9.31 events/h. Age at PSG was not significantly associated with AHI. AHI correlated with ODI, and clinically documented adenoidal hypertrophy correlated with both AHI and ODI. Males showed significantly higher ODI values than females, whereas sex differences in AHI, minimum SpO_2_, and time with SpO_2_ < 90% were not significant. In exploratory multivariable models, adenoidal hypertrophy was associated with higher ODI, while adenotonsillectomy was associated with lower minimum SpO_2_. **Conclusions**: The association between atrioventricular canal defect and hypoxaemic burden was not robust in bootstrap analyses. These exploratory findings do not warrant changes to current screening management strategies, but support further prospective studies incorporating standardised ENT and cardiac characterisation.

## 1. Introduction

Down syndrome (DS) is the most common chromosomal abnormality compatible with life; prevalence estimates at birth vary from about 1 in 800 to 1 in 1000 live births in Western countries [1]. Children with DS have a significantly increased risk of developing sleep-disordered breathing (SDB), particularly obstructive sleep apnoea (OSA), due to the combination of craniofacial features, generalised hypotonia, macroglossia, dynamic collapse of the upper airways, and frequent adeno-tonsillar hypertrophy [2,3]. Classical and contemporary studies estimate that over 50% of children with DS develop a clinically significant form of OSA, with prevalence rates much higher than those reported in the general paediatric population, where the disorder affects about 1–5% of individuals [2].

The consequences of OSA in childhood include behavioural alterations, cognitive difficulties, reduced quality of life, and potential cardiovascular complications such as hypertension and autonomic dysfunction [4,5]. In children with DS, such effects can be amplified due to the presence of common comorbidities, particularly congenital heart diseases, hypothyroidism, otorhinolaryngological disorders, and orthopaedic problems [2,6]. The interaction between anatomical, neuromuscular, and metabolic factors contributes to a highly heterogeneous nocturnal respiratory phenotype, making it challenging to identify a reliable independent association with OSA severity.

To provide an explicit a priori rationale for the clinical variables investigated, we focused on readily available features that reflect major pathophysiological domains of SDB in DS and that are commonly considered in paediatric OSA research. Age, sex, and body mass index were included as standard demographic and anthropometric variables used in paediatric OSA studies and in DS-specific analyses. However, their discriminatory value in DS remains debated [7,8,9]. ENT-related variables (adenoidal and tonsillar hypertrophy, prior adenotonsillectomy, and ongoing ENT therapy) were selected because upper-airway anatomy and lymphoid tissue hypertrophy are central contributors to obstructive events in DS and represent potentially modifiable targets [10,11]. Congenital heart disease was examined, given its high prevalence in DS and its potential to increase vulnerability to intermittent hypoxaemia and cardiopulmonary sequelae [10,12,13,14]. Finally, in addition to AHI, we analysed ODI and time with SpO_2_ < 90% (minutes) to capture desaturation frequency and hypoxaemic burden, which may be clinically relevant severity dimensions beyond event counts alone [10,12].

Paediatric guidelines emphasise the crucial importance of routine screening in DS irrespective of symptoms [4,5]. This approach stems from evidence showing that parent-reported symptoms, such as snoring or witnessed apnoeas, have a poor correlation with actual polysomnographic severity in children with DS [2]. However, despite international recommendations, clinical practice still faces significant challenges, including limited access to diagnostic testing, variability in screening timing, and uncertainty about the role of clinical variables such as age, BMI, sex, and comorbidities in predicting disease severity.

Against this background, the present study examines a cohort of children with DS who underwent full PSG to assess the relationships between specific clinical characteristics and SDB severity. The analysis allows us to determine whether variables commonly regarded as predictive in the general paediatric population—such as age or BMI—retain discriminative value in a group characterised by marked anatomical and functional heterogeneity and by a high prevalence of comorbidities. Clarifying the contribution of these factors may help the early identification of at-risk individuals and optimise screening and follow-up protocols for children with DS.

### Aims

The aim was to examine the associations between selected clinical characteristics—including age, sex, BMI, cardiac conditions, hearing disorders, and otolaryngological variables—and PSG-derived SDB severity metrics in a cohort of children with DS, with particular attention to desaturation frequency and hypoxaemic burden. The aim was to explore adjusted associations between selected clinical variables and PSG-derived severity metrics.

## 2. Materials and Methods

### 2.1. Inclusion Criteria

In total, 48 participants were included if they met all the following criteria:

- Children and adolescents with DS, up to 18 years of age, who were prescribed and subsequently underwent overnight PSG for the evaluation of suspected SDB.
- Documented medical prescription for PSG aimed at investigating symptoms suggestive of SDB (e.g., snoring, witnessed apnoeas).
- Availability of clinical history before PSG, including information on snoring, apnoeas, or other indicators of SDB.
- Completion of the Paediatric Sleep Questionnaire at the time of PSG prescription.
- Complete medical records, including:
  ○ Sex (male = 0, female = 1).
  ○ Age at the time of PSG.
  ○ DS-specific BMI-for-age curves [13] are valid for ages 2–20 years. Five children were aged < 2 years. BMI at PSG was recorded as kg/m^2^. When applicable, DS-specific age- and sex-adjusted BMI z-scores were calculated using published DS-specific growth references; however, the BMI z-score was available only for participants within the applicable age range.
  ○ Full PSG results (Apnoea–Hypopnoea Index, AHI; Oxygen Desaturation Index, ODI; minimum SpO_2_; percentage of snoring; and other standard respiratory parameters).
  ○ Current therapies before PSG (oral, inhaled, or nasal spray medications).
  ○ History of previous Ear–Nose–Throat/otorhinolaryngological (ENT) surgery (e.g., adenoidectomy, adenotonsillectomy).
  ○ Information regarding passive tobacco smoke exposure.
  ○ Available allergy testing results (e.g., skin prick tests).

ENT-related variables (adenoidal/tonsillar hypertrophy, ENT disorders/therapy, and history of adenotonsillectomy) were extracted retrospectively from routine pre-PSG clinical documentation and coded as present/absent when explicitly reported. A uniform study-specific ENT anatomical/functional assessment with standardised grading was not available across all patients.

*Definition of clinical variables (ENT)*

- Adenoidal hypertrophy: recorded as present when explicitly described as hypertrophy/enlargement on ENT assessment or imaging noted in the clinical record; absent if explicitly stated absent; otherwise not inferred.
- Tonsillar hypertrophy: recorded as present when explicitly described as hypertrophy/enlarged tonsils; otherwise not inferred.
- ENT therapy: recorded when chronic nasal/topical therapy was listed at the time of PSG referral.
- History of adenotonsillectomy: recorded when surgical history is documented.

### 2.2. Exclusion Criteria

Participants were excluded if one or more of the following applied:

- Incomplete PSG data, including missing respiratory parameters such as AHI, ODI, minimum SpO_2_ or snoring percentage.
- Missing PSQ, or PSQ not administered at the time of PSG prescription.
- Incomplete or unclear medical history regarding symptoms suggestive of SDB or ongoing medications before PSG.
- Lack of documentation on previous ENT surgery relevant to upper-airway obstruction.
- Absence of information on the interval between PSG prescription and execution.
- Missing or inconclusive allergy testing results, when required for clinical evaluation.

### 2.3. Polysomnography

Overnight polysomnography (PSG) was performed using a Healthdyne Technologies device (E-series Compumedics Alice 3, Marietta, GA, USA), as previously described [14]. Signals used for the present analysis included airflow, thoracoabdominal effort, SpO_2_, ECG, EEG/EOG/EMG, body position, and synchronised audio/video recording. Transcutaneous CO_2_ was recorded during PSG acquisition but was neither systematically extracted from the retrospective PSG reports nor included among the predefined outcomes of this analysis. Accordingly, CO_2_-derived indices are not reported.

Sleep staging, arousal scoring, and respiratory event scoring were performed according to the American Academy of Sleep Medicine (AASM) Scoring Manual, Version 2.4, 2017 [15], using paediatric criteria. Obstructive apnoea was defined as a ≥90% reduction in airflow lasting for at least two breaths in the presence of continued respiratory effort. Hypopnoea was defined as a ≥30% reduction in airflow lasting for at least two breaths and associated with either a ≥3% decrease in SpO_2_ from baseline and/or an EEG arousal. For desaturation scoring, baseline SpO_2_ was defined as the stable oxygen saturation value immediately preceding the respiratory event or desaturation. Oxygen desaturation was therefore defined as a decrease in SpO_2_ of ≥3 percentage points from this baseline value. The oxygen desaturation index (ODI3) was calculated as the number of ≥3% desaturation events from this baseline value per hour of total sleep time. The apnoea–hypopnoea index (AHI) was calculated as the number of apnoeas plus hypopnoeas per hour of total sleep time. OSA severity was classified according to AHI as mild (1 ≤ AHI ≤ 5 events/h), moderate (5 < AHI ≤ 10 events/h), or severe (AHI > 10 events/h).

### 2.4. Statistical Analysis

Statistical analyses were performed using IBM SPSS Statistics for Windows, version 22.0 (IBM Corp., Armonk, NY, USA). Data were exported from Google Forms in XLS format and subsequently imported into a Microsoft Excel database (Microsoft Corp., Redmond, WA, USA) for data management.

Descriptive statistics were calculated for all clinical and polysomnographic variables. Continuous variables were expressed as mean ± standard deviation (SD), and categorical variables as counts and percentages. Dichotomous variables were coded as 0/1 to facilitate interpretation of the coefficients; specifically, sex was coded as 0 = male and 1 = female. All other binary clinical variables were coded as 0 = absent/not performed and 1 = present/performed. Binary variables were reported as counts and percentages rather than as means.

Comparisons of polysomnographic indices between groups were performed using the Mann–Whitney U test. Exploratory bivariate associations between clinical characteristics and polysomnographic parameters were assessed using Kendall’s tau-b correlation coefficients. This nonparametric rank-based method was chosen because the correlation matrix included both continuous and binary-coded variables.

As a sensitivity analysis, exploratory Kendall’s tau-b correlations were also calculated using the DS-specific age- and sex-adjusted BMI z-score available for participants within the applicable age range. Because the BMI z-score was available for only 43 participants, these analyses were interpreted cautiously. They were not used to replace the main descriptive analyses based on BMI at PSG, expressed as kg/m^2^.

All tests were two-sided. To address multiple testing in the exploratory correlation analyses, *p*-values were additionally adjusted using the Benjamini–Hochberg false discovery rate (FDR) procedure, with q = 0.05. Both raw *p*-values and FDR-adjusted q-values were taken into account when interpreting the results.

To explore adjusted associations between clinical variables and PSG-derived severity metrics, we fitted exploratory multivariable linear regression models for four outcomes: AHI, ODI, minimum SpO_2_, and time spent with SpO_2_ < 90%. Candidate predictors were restricted to demographic and clinical variables available before or at the time of PSG, whereas PSG-derived variables were excluded to avoid circularity. Given the limited sample size, models were restricted to a maximum of four predictors. Candidate clinical variables were selected based on biological plausibility and prior literature on SDB in DS, with an emphasis on upper-airway/ENT features, congenital heart disease, and standard demographic and anthropometric variables used in paediatric OSA research [8,10,12,16]. Candidate models were compared using the Bayesian information criterion (BIC), and the most parsimonious model with the lowest BIC was retained for each outcome. For each final model, we reported the number of participants included, the number of predictors excluding the intercept, the N/predictor ratio, unstandardised regression coefficients (B), conventional 95% confidence intervals, *p*-values, bootstrap 95% confidence intervals based on 2000 resamples, R^2^, and diagnostic notes. Model diagnostics included assessment of multicollinearity using tolerance and VIF values, visual inspection of residual plots, and evaluation of potentially influential observations using studentised residuals, leverage values, and Cook’s distance. Given the exploratory and data-driven nature of these analyses, the models were interpreted as hypothesis-generating rather than predictive.

ENT-related variables, including adenoidal hypertrophy, tonsillar hypertrophy, ENT therapy, and previous adenotonsillectomy, were extracted retrospectively from routine pre-PSG clinical documentation and coded as present or absent only when explicitly reported. These variables were not derived from a uniform study-specific ENT protocol. No standardised grading of adenoidal or tonsillar size was available, and relevant nasal or upper-airway structures, including turbinate hypertrophy and septal deviation, were not systematically assessed across all participants. Moreover, no standardised functional assessment of upper-airway obstruction was available. Therefore, ENT variables should be interpreted as crude clinical indicators of documented ENT findings rather than as quantitative anatomical or functional measures of airway obstruction.

For the age–AHI analysis, the same complete-case sample was used for both Kendall’s tau-b correlation and the univariate regression analysis (*n* = 48). The same age variable, age at PSG expressed in years, was used in both analyses.

## 3. Results

Table 1 presents descriptive statistics for the main continuous clinical and polysomnographic variables in the analysed sample of 48 children and adolescents with DS, of whom 29 were male (60.4%). The mean age at PSG was 6.29 ± 3.98 years, and the mean BMI at PSG was 18.60 ± 4.80 kg/m^2^. Regarding PSG parameters, the mean AHI was 10.51 ± 11.82 events/h, with considerable variability, reaching a maximum of 49.8 events/h. Mean ODI was 7.07 ± 9.31 events/h, with a maximum of 42.4 events/h. Time spent with SpO_2_ < 90% was highly variable, with a median of 0.65 min and a maximum of 99 min. OSA prevalence was 93.8% for AHI ≥ 1 event/h; moderate-to-severe OSA (AHI ≥ 5 events/h) was present in 58.3%, and 35.4% had AHI ≥ 10 events/h.

Table 2 reports categorical and binary clinical variables as counts and percentages. Cardiac pathologies were present in 37/48 participants (77.1%), whereas hearing disorders were reported in 11/48 participants (22.9%). Otorhinolaryngological disorders were recorded in 33/48 participants (68.8%), tonsillar hypertrophy in 28/48 (58.3%), and adenoidal hypertrophy in 20/48 (41.7%).

**Figure 1:**
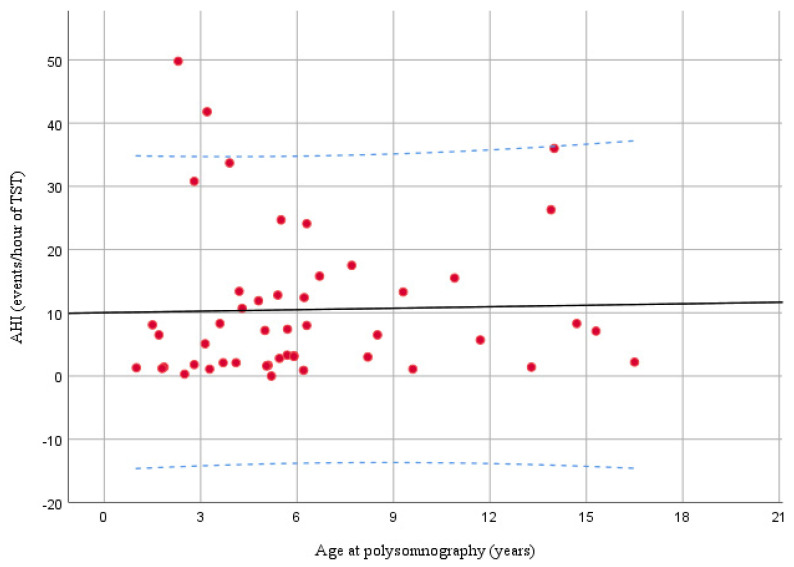
Scatterplot showing the association between age at polysomnography (age at PSG, years) and AHI in the complete-case sample (*n* = 48), with univariate linear regression line and 95% confidence interval (blue dashed line). The model explained negligible variance in AHI (R^2^ = 0.001; *p* = 0.859).

Figure 1 shows the association between age at polysomnography (age at PSG, years) and AHI in the complete-case sample of 48 participants. No significant association was observed. The univariate linear regression model explained negligible variance in AHI (R^2^ = 0.001; *p* = 0.859). The same age variable, units, sample size, and exclusion criteria were used for both analyses.

**Figure 2:**
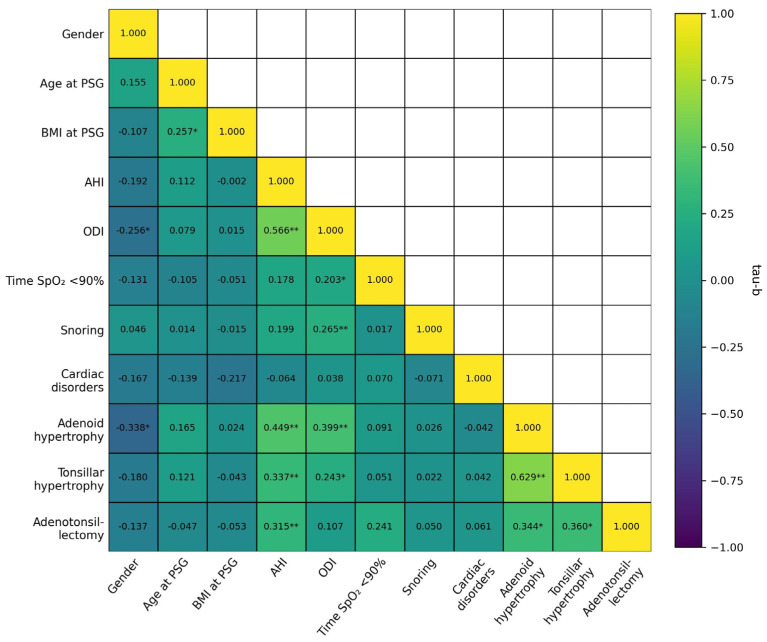
An exploratory Kendall’s tau-b correlation matrix in 48 children with Down syndrome. Units: age at PSG (years), BMI at PSG (kg/m^2^), AHI and ODI (events/h of total sleep time), time with SpO_2_ < 90% (minutes), and snoring (% of total sleep time). Sex was coded as 0 = male, 1 = female. Binary clinical variables were coded as 0 = no/not performed, 1 = yes/performed and included cardiac disorders, adenoidal hypertrophy, tonsillar hypertrophy, and adenotonsillectomy. * *p* < 0.05; ** *p* < 0.01.

Figure 2 shows the exploratory Kendall’s tau-b correlation matrix for age at PSG, sex, BMI, polysomnographic parameters, and the main clinical comorbidities. The most robust correlations were observed between AHI and ODI (τ = 0.566; *p* < 0.001), between adenoidal hypertrophy and AHI (τ = 0.449; *p* < 0.001), between adenoidal hypertrophy and ODI (τ = 0.399; *p* = 0.001), and between adenoidal hypertrophy and tonsillar hypertrophy (τ = 0.629; *p* < 0.001). After Benjamini–Hochberg FDR adjustment, the remaining significant associations were AHI with ODI, adenoidal hypertrophy with AHI, adenoidal hypertrophy with ODI, and adenoidal hypertrophy with tonsillar hypertrophy. Other nominal associations were interpreted cautiously as exploratory.

In a sensitivity analysis restricted to participants with available DS-specific BMI z-score data (*n* = 43), BMI z-score was likewise not significantly associated with AHI (τ = 0.030; *p* = 0.777), ODI (τ = 0.064; *p* = 0.544), minimum SpO_2_ (τ = −0.122; *p* = 0.257), or time spent with SpO_2_ < 90% (τ = 0.006; *p* = 0.958). These analyses were considered exploratory because the BMI z-score was not available for the five children aged < 2 years.

**Figure 3:**
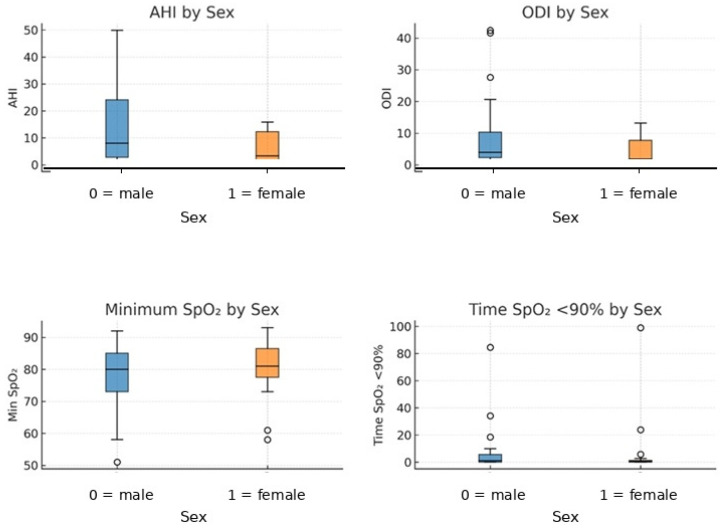
Boxplots of polysomnographic parameters by sex (0 = male, 1 = female). Panels show AHI, ODI, minimum SpO_2_, and time with SpO_2_ < 90%. Mann–Whitney U test was used. ODI was higher in males (*p* = 0.034); AHI, minimum SpO_2_, and time with SpO_2_ < 90% showed no significant differences.

Figure 3 illustrates the distribution of polysomnographic parameters stratified by sex, coded as 0 = male and 1 = female. In Mann–Whitney U tests, the distribution of AHI did not differ significantly between males and females (*p* = 0.111). ODI differed significantly by sex, with higher values in males than in females (*p* = 0.034). No significant sex differences were observed for minimum SpO_2_ (*p* = 0.512) or for time spent with SpO_2_ < 90% (*p* = 0.289).

Supplementary Table S1 reports the exploratory multivariable regression models for PSG-derived severity outcomes. All models included 48 participants and had a maximum of four predictors, with N/predictor ratios ranging from 16.0 to 24.0. PSG-derived variables were not included as candidate predictors to avoid circularity. Overall, the selected models explained a modest proportion of outcome variance, with R^2^ values ranging from 0.229 for AHI to 0.367 for minimum SpO_2_.

For AHI, the selected model included patent foramen ovale and adenoidal hypertrophy and explained 22.9% of the variance. Adenoidal hypertrophy was associated with higher AHI (B = 10.840, 95% CI 4.449 to 17.230; *p* = 0.001; bootstrap 95% CI 4.942 to 17.080), whereas the association with patent foramen ovale was borderline and not clearly supported by the conventional confidence interval crossing zero (B = 6.661, 95% CI −0.135 to 13.458; *p* = 0.055).

For ODI, the selected model included adenoidal hypertrophy and ENT therapy and explained 26.4% of the variance. Adenoidal hypertrophy was associated with higher ODI (B = 6.414, 95% CI 1.271 to 11.557; *p* = 0.016; bootstrap 95% CI 1.541 to 12.653). ENT therapy also showed a positive conventional association with ODI (B = 6.619, 95% CI 0.123 to 13.114; *p* = 0.046), although the bootstrap confidence interval crossed zero, indicating limited robustness.

For the lowest SpO_2_, the selected model included adenotonsillectomy, coeliac disease, and genitourinary abnormalities and explained 36.7% of the variance. Adenotonsillectomy was associated with lower minimum SpO_2_ (B = −9.965, 95% CI −15.674 to −4.256; *p* = 0.001; bootstrap 95% CI −16.003 to −4.185). Coeliac disease was associated with higher minimum SpO_2_ (B = 7.920, 95% CI 2.627 to 13.212; *p* = 0.004; bootstrap 95% CI 3.279 to 13.384). Genitourinary abnormalities showed a borderline conventional association (B = 5.795, 95% CI −0.138 to 11.728; *p* = 0.055), although the bootstrap confidence interval did not cross zero; this finding should therefore be interpreted cautiously.

For time spent with SpO_2_ < 90%, the selected model included atrioventricular canal defect and orthopaedic problems and explained 30.5% of the variance. Atrioventricular canal defect showed a strong positive conventional association with time spent below 90% saturation (B = 29.019, 95% CI 14.651 to 43.387; *p* < 0.001), but the bootstrap confidence interval crossed zero and influence diagnostics indicated substantial sensitivity to influential observations. Orthopaedic problems were associated with lower time spent with SpO_2_ < 90% (B = −10.568, 95% CI −20.383 to −0.753; *p* = 0.035; bootstrap 95% CI −19.685 to −1.130).

Full details of the exploratory BIC-selected multivariable regression models, including N, number of predictors, N/predictor ratios, unstandardised coefficients, conventional and bootstrap 95% confidence intervals, *p*-values, R^2^, and diagnostic notes, are provided in Supplementary Table S1.

Across models, no relevant multicollinearity was detected, with VIF values close to one. However, residual and influence diagnostics identified potentially influential observations, particularly in the ODI and time SpO_2_ < 90% models. Therefore, these findings should be interpreted as exploratory adjusted associations rather than as robust independent predictors or clinically actionable risk-stratification tools.

## 4. Discussion

In our sample of 48 children and adolescents with DS, OSA was ubiquitous (AHI ≥ 1/h in 93.8% of participants), with a substantial proportion showing moderate-to-severe forms (AHI ≥ 5/h in 58.3% and AHI ≥ 10/h in 35.4%). Although the mean BMI was only slightly above the threshold for overweight (18.6 kg/m^2^), the average AHI was approximately 10.5 events per hour, with wide interindividual variability and peak values reaching 50 events/h. ODI and oxygenation parameters also displayed considerable dispersion, with minimum SpO_2_ values as low as 51% and time spent with SpO_2_ < 90% (minutes) reaching nearly 100 min in some cases. The respiratory picture emerged within a context of pronounced multimorbidity: more than three-quarters of patients had congenital heart disease, almost 70% had ENT-related problems, about half had visual disorders, and over one-third presented with tonsillar hypertrophy, adenoidal hypertrophy, hypothyroidism or other systemic comorbidities, reflecting the clinical complexity typically observed in individuals with DS.

We acknowledge that several findings of this study are confirmatory rather than novel. In particular, the high burden of OSA/SDB in children with DS, the limited discriminatory value of basic demographic or anthropometric variables, and the relevance of ENT and cardiac comorbidities have already been reported in previous DS cohorts and reviews. Accordingly, the present study should not be interpreted as proposing a new predictive model, mechanistic framework, or clinical decision pathway. Its contribution is more limited and exploratory: it provides cohort-specific descriptive and hypothesis-generating data on associations between routinely recorded clinical features and multiple PSG-derived severity domains, including desaturation frequency and hypoxaemic burden. The present study is therefore best positioned as a preliminary, hypothesis-generating clinical cohort analysis rather than as evidence for validated predictors or immediate changes to screening, diagnostic, or management strategies.

Given the limited sample size, the multivariable analyses were deliberately restricted to exploratory models with no more than four predictors. Candidate predictors were limited to demographic and clinical variables, and PSG-derived variables were excluded to avoid circularity. Although the selected models suggested clinically plausible associations, including adenoidal hypertrophy with AHI/ODI, adenotonsillectomy with minimum SpO_2_, and atrioventricular canal defect with time spent with SpO_2_ < 90%, residual and influence diagnostics indicated potentially influential observations in some models. Therefore, these findings should be interpreted as exploratory and hypothesis-generating rather than as robust independent predictors or clinically actionable risk-stratification tools.

Two core observations from our cohort should be interpreted as confirmatory. First, the exceptionally high prevalence of OSA and the substantial proportion of moderate-to-severe disease are consistent with prior DS cohorts and reviews, which describe OSA as a near-ubiquitous comorbidity in childhood DS [9,10]. Second, basic demographic and anthropometric features (age, sex, and BMI) showed limited discriminatory value for PSG-defined severity when considered in isolation, in line with DS-focused studies and meta-analytic evidence that clinical phenotyping is an imperfect proxy for PSG severity [8,9,16]. The incremental contribution of the present study is to quantify these associations in multivariable models across complementary PSG domains, including desaturation frequency and hypoxaemic burden.

The analysis of associations showed that age plays a modulatory role, although it is not sufficient on its own to account for OSA severity. Males tended to have higher AHI and ODI values than females, although only the difference in ODI reached statistical significance. In the exploratory multivariable models, adenoidal hypertrophy was associated with higher AHI and ODI, whereas adenotonsillectomy was associated with lower minimum SpO_2_. Atrioventricular canal defect showed a positive conventional association with time spent with SpO_2_ < 90%; however, this association was not robust in bootstrap analysis and appeared sensitive to influential observations. Therefore, these findings should be interpreted as exploratory signals rather than as robust independent predictors.

In our cohort, raw BMI (kg/m^2^) was not associated with AHI, ODI, minimum SpO_2_, or time spent with SpO_2_ < 90%. A sensitivity analysis using DS-specific age- and sex-adjusted BMI z-scores in the 43 participants for whom these data were available yielded similar null findings. However, these results should be interpreted conservatively. The wide paediatric age range, the absence of BMI z-score data for children younger than 2 years, and the limited sample size reduce the ability to draw firm conclusions regarding the role of adiposity in DS-related SDB. Therefore, the present findings should not be taken as evidence against a possible contribution of adiposity, but rather as an indication that anthropometric effects were not clearly detectable in this retrospective cohort.

In the general paediatric population, the overall prevalence of OSA is approximately 1–4%, whereas moderate-to-severe forms (AHI ≥ 5/h) likely affect fewer than 1% of individuals, based on available population studies [17,18]. Therefore, the frequency of SDB observed in our cohort appears to be significantly higher compared to epidemiological data from the non-DS population, emphasising the importance of considering DS as a primary risk factor for severe clinical presentations.

The wide variability observed in PSG parameters (AHI, ODI, and minimum SpO_2_) suggests the presence of distinct OSA phenotypes within the DS population [16]. Previous studies have described, in patients with DS, both predominantly obstructive respiratory patterns and cases with central and mixed apneas [19,20], attributable to factors such as oropharyngeal hypotonia, macroglossia, and adeno-tonsillar hypertrophy [16,21,22]. In particular, the absence of macroscopic nasal or tonsillar obstructions, as observed in some cases, did not prevent the detection of very high AHI peaks, confirming that the sole anatomical assessment is insufficient to predict the severity of OSA in children with SDB [8,16]. These different phenotypic elements require targeted diagnostic investigation (e.g., advanced airway imaging or functional studies) to clarify the underlying causes and prepare personalised treatment strategies [23,24].

In line with previous studies, age and anthropometric variables such as BMI did not reliably predict moderate-to-severe OSA in children with DS. However, because BMI was analysed as an absolute kg/m^2^ value rather than as an age- and sex-standardised measure (ideally using DS-specific references), this null association should be interpreted conservatively. We cannot exclude that standardised adiposity indices might show different relationships with AHI/ODI and oxygenation metrics [2,25]. In contrast, anatomical factors and altered respiratory control appear to play a more prominent role [2,9,16]. This is because, in this population, anatomical factors (e.g., lingual hypotonia and oropharyngeal structures) and alterations in central respiratory control may outweigh the impact of body weight [10,20]. Consistently, current guidelines from the American Academy of Paediatrics recommend routine polysomnographic screening for all children with DS by 4 years of age, regardless of symptoms or standard anthropometric parameters [26,27,28,29].

In this study, the most frequent comorbidities were cardiovascular conditions (77.1%) and ENT disorders (68.8%), with notable incidences of visual impairments (50.0%), tonsillar hypertrophy (58.3%), and hypothyroidism (41.7%). These findings are consistent with the existing literature, which highlights how congenital heart diseases and endocrine–metabolic imbalances increase the risk of complications in SDB among individuals with DS [10,12,30]. In particular, adeno-tonsillar hypertrophy is an organic factor often associated with increased upper-airway obstruction [8,11]. The high prevalence of orthopaedic conditions (37.5%) and coeliac disease (33.3%) further supports the view that early multidisciplinary involvement—including cardiology, endocrinology, ENT, orthopaedics, and ophthalmology—is essential to optimise the diagnostic and therapeutic pathway [11,30]. In the literature, adenotonsillectomy is associated with an improvement in polysomnographic parameters and quality of life [31,32,33,34]. In our sample, however, the children who had already undergone surgery had lower minimum SpO_2_ levels, presumably because they represented cases with more severe respiratory conditions.

The association between clinically documented adenoidal hypertrophy and higher AHI/ODI should be interpreted cautiously. Because ENT variables were retrospectively extracted from routine clinical records and were not based on standardised anatomical grading or functional airway assessment, this finding cannot establish that adenoidal obstruction per se was the causal mechanism underlying increased respiratory event frequency or desaturation burden [10]. Rather, it suggests that documented ENT abnormalities may co-occur with more severe PSG-derived respiratory indices in this cohort. This observation should be considered confirmatory and hypothesis-generating, not mechanistically novel, and requires confirmation in prospective studies with standardised ENT phenotyping, including graded assessment of adenoidal and tonsillar size, systematic evaluation of nasal structures, and, where feasible, functional assessment of upper-airway obstruction.

Emerging evidence indicates that, in addition to anatomical alterations (e.g., macroglossia, reduction in pharyngeal diameters) [10,35,36], neuromuscular alterations and dysfunctions of the respiratory control centre occur. In particular, the widespread hypotonia typical of DS reduces the tone of the dilator muscles of the upper airways during sleep, favouring obstructive collapse [10,36,37]. At the same time, forms of central apneas have been documented in subjects with DS, suggesting a deficit in ventilatory control [20,36,38]. Furthermore, hypothyroidism can further contribute to reduced muscle tone and ineffective ventilation, worsening the severity of SDB [39,40]. These pathophysiological features, which are less typical of classic paediatric OSA, support the need for a diagnostic and therapeutic approach tailored to children with DS.

Our findings are consistent with existing recommendations supporting systematic PSG-based screening in children with DS, given the high prevalence and heterogeneity of SDB in this population [2,26,41]. However, because the present study is retrospective, exploratory, and based on a limited sample size, our data should not be interpreted as sufficient to define new risk algorithms or clinical prioritisation pathways [9,16,42]. Rather, they support the need for larger prospective studies to integrate clinical, ENT, cardiological, and PSG-derived variables into validated DS-specific risk assessment frameworks.

Timely identification of SDB remains important because untreated SDB may contribute to daytime fatigue, neurocognitive difficulties, and long-term cardiovascular complications. However, the modest explanatory power of the exploratory models in the present study indicates that routinely recorded clinical variables capture only part of the variability in PSG-derived outcomes. This supports the need for multidisciplinary follow-up and prospective studies integrating clinical, ENT, cardiological, and PSG-derived variables, rather than reliance on any single clinical marker [11,43,44].

In children with DS, atrioventricular septal defect (AVC)—a form of defect characterised by a left-to-right shunt and pulmonary volume overload—is one of the most typical cardiac anomalies and represents a significant risk factor for the early development of pulmonary hypertension, driven by increased pulmonary blood flow, endothelial shear stress, and vascular remodelling [45,46]. Furthermore, OSA and intermittent hypoxaemia are common comorbidities and are recognised as independent contributors to the pulmonary pressure burden [47,48,49]. In this context, it is plausible that, in children with DS and AVC, the combination of an intracardiac shunt, possible pulmonary hypertension, and increased vulnerability of the vascular bed makes each nocturnal desaturation episode more profound and more prolonged, ultimately leading to more time spent with SpO_2_ < 90%. However, no studies have specifically assessed atrioventricular canal defects as an independent factor associated with nocturnal hypoxaemic burden in this population.

Regarding oxygenation parameters, atrioventricular canal defect showed a positive association with time spent with SpO_2_ < 90% in the conventional regression model. However, the bootstrap confidence interval crossed zero, and influence diagnostics suggested sensitivity to influential observations. Therefore, this finding should be regarded as hypothesis-generating. Although the association is biologically plausible in the context of congenital heart disease, pulmonary vascular vulnerability, and intermittent hypoxaemia in DS, it should not be interpreted as evidence that atrioventricular canal defect independently identifies a subgroup requiring different clinical management. Prospective studies specifically designed to evaluate cardiac phenotype, pulmonary haemodynamics, and nocturnal oxygenation are needed before any clinical prioritisation can be inferred.

The association between a history of adenotonsillectomy and lower minimum SpO_2_ is likely influenced by the selection of children with more severe baseline disease. In the exploratory model for minimum SpO_2_, adenotonsillectomy was associated with lower minimum SpO_2_ values (B = −9.965, 95% CI −15.674 to −4.256; *p* = 0.001; bootstrap 95% CI −16.003 to −4.185; model R^2^ = 0.367). This association should not be interpreted as a harmful effect of surgery itself, but rather as a possible example of confounding by indication.

Children selected for surgery tend to be those with more severe baseline respiratory impairment, complex respiratory phenotypes, or multilevel obstruction [37], and presumably maintain a residual hypoxaemic load despite the reduction in AHI after adenotonsillectomy [34]. This is in line with the literature, which documents partial improvement of OSA after AT, with persistence of moderate-to-severe OSA and gas exchange abnormalities in a high proportion of patients [50]. This finding underscores that surgery does not guarantee complete resolution of the condition and that in children with DS who undergo adenotonsillectomy, it is advisable to maintain polysomnographic follow-up over time.

In our cohort, raw BMI was not associated with overall OSA severity (AHI/ODI), consistent with previous studies showing the limited predictive value of anthropometric measures for paediatric OSA in DS [9]. In contrast, BMI was significantly associated with the number of mixed and central apnoeas—a finding that has not previously been described and should be interpreted with caution. One possible explanation is that, in individuals with DS, the contribution of BMI to the pathophysiology of SDB may manifest primarily through hypoventilation and instability of ventilatory control. This is consistent with studies linking higher BMI to an increased risk of nocturnal hypercapnia and hypoventilation in this population [21]. This combination of obesity-related mechanical load and the inherent vulnerability of the respiratory control centre, typical of DS, may favour an increase in mixed and central events, rather than a linear rise in obstructive AHI [51]. However, this result requires confirmation in larger cohorts.

Taken together, our findings suggest that routinely recorded ENT findings, selected cardiac comorbidities, and PSG-derived oxygenation metrics may provide complementary information when describing SDB heterogeneity in children with DS. However, given the retrospective design, limited sample size, non-standardised ENT assessment, and exploratory model selection, these variables should not be interpreted as validated clinical predictors or as sufficient to modify current screening, diagnostic, or management strategies. Their main value lies in supporting the design of larger prospective studies with standardised anatomical and functional airway assessment and predefined PSG-derived outcomes.

### Strengths and Limitations

The retrospective design and the relatively small sample size represent the main limitations of this study, as does the absence of a direct comparison with a non-DS control group. Given the limited sample size relative to the number of candidate covariates, coefficient estimates may be unstable, and overfitting cannot be excluded.

Although the exploratory regression models were restricted to a maximum of 4 predictors, the final BIC-selected models included 2 or 3 predictors, with N/predictor ratios ranging from 16.0 to 24.0. Despite this parsimonious approach, the sample size remains small for adjusted modelling, and residual and influence diagnostics suggested potentially influential observations in some models. Therefore, even associations supported by conventional or bootstrap confidence intervals should be interpreted as exploratory and hypothesis-generating rather than as robust independent predictors.

ENT variables represent a major methodological limitation of the present study. They were coded from routine clinical records as binary present/absent variables and lacked standardised anatomical grading or functional characterisation. In particular, nasal and upper-airway structures such as turbinate hypertrophy and septal deviation were not systematically evaluated, and no functional assessment of airway collapsibility or obstruction was available. Consequently, associations between ENT variables and PSG outcomes should be interpreted with caution and cannot be used to infer the anatomical site, severity, or mechanism of upper-airway obstruction. Although several studies have examined associations between sleep disorders in children with DS and daytime functioning, behaviour, and cognitive performance [43,52,53], the available literature remains relatively limited and is often based on small, predominantly cross-sectional samples [12,54]. In the present study, the retrospective and non-standardised nature of ENT data collection prevents inference of a direct anatomical mechanism or guidance for ENT management [12,53]. These exploratory findings do not modify current screening or management strategies; rather, they suggest that ENT and cardiac characterisation may be useful variables to include in future prospective studies of PSG-derived oxygenation burden in children with DS.

A further limitation concerns the assessment of nutritional status. Although BMI at PSG was recorded for all participants, raw BMI (kg/m^2^) is difficult to interpret in a paediatric cohort with a wide age range. DS-specific age- and sex-adjusted BMI z-scores were available for 43 participants, but these data were not available for the five children younger than 2 years. Accordingly, conclusions regarding the role of anthropometric factors remain limited. Future studies should use DS-specific age- and sex-standardised adiposity measures throughout the full paediatric age range and, where feasible, incorporate direct measures of body composition.

Conversely, several methodological aspects strengthen the validity of our findings. A significant strength is the use of a full, standardised PSG protocol applied uniformly to all participants, ensuring high data quality and allowing robust comparisons across subjects [41,51], in line with current recommendations that identify overnight PSG as the diagnostic gold standard for SDB in children with DS [10]. CO_2_-derived PSG variables were not systematically available for retrospective analysis and were therefore not included. Consequently, this study cannot assess nocturnal hypoventilation or hypercapnia in this cohort.

In addition, the comprehensive clinical characterisation of the cohort—including detailed cardiological, otorhinolaryngological, endocrine, metabolic and other systemic comorbidities—enabled multivariable analyses adjusted for multiple potential confounders [16]. The integration of clinical variables with objective sleep metrics provided a nuanced picture of the determinants of SDB severity in children with DS [12]. Exploratory multivariable analyses suggested adjusted associations between selected clinical variables and PSG-derived severity metrics, such as adenoidal hypertrophy with AHI/ODI and adenotonsillectomy with minimum SpO_2_. However, these findings require confirmation in larger cohorts before being used for formal risk stratification. Finally, the focus on a high-risk yet relatively understudied population, coupled with a multidimensional analytical approach that reflects real-world clinical complexity, enhances the translational value and applicability of the results.

Future studies should adopt a prospective design with standardised anatomical/functional ENT assessment (including nasal structures and graded endoscopic evaluation) and larger cohorts to enable robust multivariable modelling and external validation. Overall, the combination of small sample size, retrospective data collection, non-standardised ENT assessment, and confirmatory findings limits the originality and external validity of the present work. The results should therefore be regarded as preliminary and hypothesis-generating.

## 5. Conclusions

In our cohort of children with DS, SDB was extremely common and often severe, with a very high prevalence of OSA and a substantial nocturnal hypoxaemic burden in a considerable proportion of participants. Basic demographic and anthropometric variables (age, sex, and raw BMI expressed as absolute kg/m^2^) accounted for only a limited share of the variability in polysomnographic parameters. Importantly, because BMI was not age- and sex-standardised (and DS-specific references were not applied), the lack of an observed BMI–AHI/ODI association in this cohort may be methodological and should be interpreted cautiously, without excluding a potential role of adiposity. An exploratory correlation between raw BMI and indices of mixed/central apnoea was observed; this should be interpreted cautiously and warrants confirmation. In contrast, adenoidal hypertrophy was associated with increased ODI in exploratory models. Atrioventricular canal defect was associated with a conventional pattern of increased time spent with SpO_2_ < 90%, but this finding was not robust in bootstrap analysis and should be interpreted cautiously.

Overall, our results indicate that routinely documented ENT findings, specific cardiac comorbidities, and PSG oxygenation metrics can offer complementary insights into the heterogeneity of SDB in children with DS. However, given the retrospective design, limited sample size, non-standardised ENT assessment, and exploratory model selection, these observations should be interpreted as preliminary and hypothesis-generating. They should not be considered validated clinical predictors, causal associations, or evidence sufficient to modify current screening or management strategies. Prospective studies with larger cohorts, standardised airway assessment, DS-specific anthropometric measures, and predefined PSG outcomes are needed before these findings can inform formal clinical decision-making.

## Figures and Tables

**Table 1:** Descriptive statistics of clinical and polysomnographic variables in the study sample *n* = (48 participants with DS).

Variable	Mean	S.D.	Min	25th Percentile	Median	75th Percentile	Max
Age at PSG (years)	6.29	3.98	1	3.36	5.43	8.09	16.5
BMI at PSG	18.60	4.80	13.8	15.35	16.80	20.35	36.2
BMI z-score at PSG (*n* = 43)	−0.16	1.40	−2.29	−1.07	−0.19	0.35	4.69
AHI (events/h)	10.51	11.82	0.0	1.86	6.80	13.38	49.8
ODI (events/h)	7.07	9.31	0.0	1.40	3.25	9.88	42.4
Snoring (%)	7.29	8.54	0.0	0.18	4.25	12.63	33.0
Obstructive apnea (events/h)	6.68	8.92	0.0	0.20	1.75	11.60	27.0
Mixed apnea (events/h)	0.09	0.35	0.0	0.0	0.0	0.00	2.0
Central apnea (events/h)	0.99	1.72	0.0	0.0	0.20	0.95	8.0
Hypopnoea (events/h)	3.12	4.72	0.0	0.25	1.20	4.95	23.4
Mean SpO_2_ (%)	94.93	3.20	81.2	94.0	96.0	97.00	99.0
Minimum SpO_2_ (%)	78.71	9.97	51	73.5	80.0	85.75	93.0
SpO_2_ level below 90%	6.93	19.09	0.0	0.10	0.650	5.28	99.0

Abbreviations: AHI = apnea–hypopnoea index; BMI = body mass index; Mean SpO_2_ = mean nocturnal oxygen saturation; minimum SpO_2_ = lowest oxygen saturation recorded during PSG; ODI = oxygen desaturation index; SpO_2_ = peripheral oxygen saturation; time SpO_2_ < 90% = time in minutes with oxygen saturation below 90%.

**Table 2:** Distribution of categorical and binary clinical variables in the study sample. Data are reported as absolute numbers and percentages. Sex was coded as 0 = male and 1 = female for statistical analyses; binary clinical variables were coded as 0 = absent/not performed and 1 = present/performed.

Variables	Count (*n*)	Percentage (%)
Sex (males)	29	60.4
Cardiac disorders	37	77.1
Otorhinolaryngological disorders	33	68.8
Tonsillar hypertrophy	28	58.3
Vision disorder	24	50.0
Adenoidal hypertrophy	20	41.7
Hypothyroidism	20	41.7
Orthopaedic problems	18	37.5
Coeliac disease	16	33.3
Patent foramen ovale	15	31.3
Ventricular septal defect	11	22.9
Hearing disorder	11	22.9
Adenotonsillectomy	11	22.9
Genito-urinary abnormalities	11	22.9
Atrial septal defect	10	20.8
Gastrointestinal pathologies	13	27.1
Persistent ductus arteriosus	9	18.8
ENT therapy	9	18.8
Airways disease	9	18.8
Haematological disorder	9	18.8
Complex congenital heart disease	12	25.0
Urinary/-ehavioural problems	7	14.6
Atrioventricular canal defect	6	12.5
Metabolic problem	6	12.5

## Data Availability

The original contributions presented in this study are included in the article/Supplementary Materials. Further inquiries can be directed to the corresponding author.

## Supplementary Materials

The following supporting information can be downloaded at: https://www.mdpi.com/article/10.3390/jcm15145581/s1, Table S1. Exploratory BIC-selected multivariable linear regression models for PSG-derived severity outcomes.

## Abbreviations

AHI	Apnoea–Hypopnoea Index
DS	Down syndrome
ENT	Ear–Nose–Throat/otorhinolaryngological
OSA	Obstructive Sleep Apnoea
ODI	Oxygen Desaturation Index
PSG	Polysomnography
SDB	Sleep-Disordered Breathing
SpO_2_	Peripheral Oxygen Saturation
