# Supplementary material for: Understanding Severe Sleep-Disordered Breathing in Down Syndrome: Insights from a Clinical–Polysomnographic Cohort
Source: J Clin Med. 2026 Jul 16;15(14):5581. doi: 10.3390/jcm15145581 (PMC13413118; doi:10.3390/jcm15145581)
Supplement: Supplementary file 1 [file jcm-15-05581-s001.zip › jcm-4080365-supplementary.pdf]

Supplementary Table S1. Exploratory BIC-selected multivariable linear regression models for PSG-derived severity outcomes (N = 48).

Outcome	Predictor	N	k predictors	N/k	B	95% CI for B	p-value	Bootstrap 95% CI	R^2	Diagnostic notes
**AHI**	Patent foramen ovale	48	2	24.0	6.661	−0.135 to 13.458	0.055	−0.441 to 14.436	0.229	VIF 1.04–1.04; max studentized deleted residual 4.63; max Cook's D 0.388; 2 cases with Cook's D >4/N.
	Adenoidal hypertrophy	48	2	24.0	10.840	4.449 to 17.230	0.001	4.942 to 17.080	0.229	Same model diagnostics.
**ODI**	Adenoidal hypertrophy	48	2	24.0	6.414	1.271 to 11.557	0.016	1.541 to 12.653	0.264	VIF 1.14–1.14; max studentized deleted residual 5.06; max Cook's D 0.510; 5 cases with Cook's D >4/N.
	ENT therapy	48	2	24.0	6.619	0.123 to 13.114	0.046	−1.419 to 16.504	0.264	Same model diagnostics.
**Minimum SpO_2**	Adenotonsillectomy	48	3	16.0	−9.965	−15.674 to −4.256	0.001	−16.003 to −4.185	0.367	VIF 1.01–1.09; max studentized deleted residual 3.70; max Cook's D 0.149; 3 cases with Cook's D >4/N.
	Coeliac disease	48	3	16.0	7.920	2.627 to 13.212	0.004	3.279 to 13.384	0.367	Same model diagnostics.
	Genitourinary abnormalities	48	3	16.0	5.795	−0.138 to 11.728	0.055	0.932 to 11.371	0.367	Same model diagnostics.
**Time SpO_2 <90%**	Atrioventricular canal defect	48	2	24.0	29.019	14.651 to 43.387	<0.001	−3.217 to 67.653	0.305	VIF 1.01–1.01; max studentized deleted residual 5.50; max Cook's D 1.425; 6 cases with Cook's D >4/N and 6 with high leverage.
	Orthopaedic problems	48	2	24.0	−10.568	−20.383 to −0.753	0.035	−19.685 to −1.130	0.305	Same model diagnostics.

Exploratory multivariable linear regression models for PSG-derived severity outcomes. Candidate predictors were restricted to demographic and clinical variables; PSG-derived variables were excluded to avoid circularity. N = number of participants included in the model; k = number of predictors excluding the intercept; N/k = number of participants per predictor. B values are unstandardised regression coefficients. Bootstrap confidence intervals were calculated using 2000 resamples. Model diagnostics included assessment of multicollinearity using VIF, residual diagnostics, studentized deleted residuals, Cook's distance, and leverage values. Because the models were exploratory and data-driven, results should be interpreted as hypothesis-generating.
